# A Comparative Analysis of XGBoost and Neural Network Models for Predicting Some Tomato Fruit Quality Traits from Environmental and Meteorological Data

**DOI:** 10.3390/plants13050746

**Published:** 2024-03-06

**Authors:** Oussama M’hamdi, Sándor Takács, Gábor Palotás, Riadh Ilahy, Lajos Helyes, Zoltán Pék

**Affiliations:** 1 Institute of Horticultural Sciences, Hungarian University of Agriculture and Life Sciences, Páter K. Str. 1, 2100 Gödöllö, Hungary; 2 Doctoral School of Plant Science, Hungarian University of Agriculture and Life Sciences, Páter K. Str. 1, 2100 Gödöllö, Hungary; 3 Univer Product Zrt, Szolnoki út 35, 6000 Kecskemét, Hungary; 4 Laboratory of Horticulture, National Agricultural Research Institute of Tunisia (INRAT), University of Carthage, Ariana 1004, Tunisia

**Keywords:** tomato quality, extreme gradient boosting, artificial neural network, prediction, shapley additive explanations

## Abstract

The tomato as a raw material for processing is globally important and is pivotal in dietary and agronomic research due to its nutritional, economic, and health significance. This study explored the potential of machine learning (ML) for predicting tomato quality, utilizing data from 48 cultivars and 28 locations in Hungary over 5 seasons. It focused on °Brix, lycopene content, and colour (a/b ratio) using extreme gradient boosting (XGBoost) and artificial neural network (ANN) models. The results revealed that XGBoost consistently outperformed ANN, achieving high accuracy in predicting °Brix (R² = 0.98, RMSE = 0.07) and lycopene content (R² = 0.87, RMSE = 0.61), and excelling in colour prediction (a/b ratio) with a R² of 0.93 and RMSE of 0.03. ANN lagged behind particularly in colour prediction, showing a negative R² value of −0.35. Shapley additive explanation’s (SHAP) summary plot analysis indicated that both models are effective in predicting °Brix and lycopene content in tomatoes, highlighting different aspects of the data. SHAP analysis highlighted the models’ efficiency (especially in °Brix and lycopene predictions) and underscored the significant influence of cultivar choice and environmental factors like climate and soil. These findings emphasize the importance of selecting and fine-tuning the appropriate ML model for enhancing precision agriculture, underlining XGBoost’s superiority in handling complex agronomic data for quality assessment.

## 1. Introduction

Tomato (*Solanum lycopersicum* L.), as one of the world’s paramount vegetable crops, is an important component of the global diet. It is one of the focal points of agronomic research due to its nutritional, economic, and health significance, and is also recognized for its culinary versatility, since the fruits are an abundant source of nutrients and bioactive compounds [1,2,3,4,5]. During the ripening process of tomatoes, a series of dramatic changes in metabolic pathway activities occur, fundamentally shaping the appearance and internal quality of the fruit [6]. Among the pivotal attributes of tomatoes that result from these alterations are their Brix value, lycopene content, and the indicative fruit colour.

The Brix degree (°Brix) is a prominent indicator of soluble solids content, mainly representing sugar concentration in juice. High °Brix values typically correspond to a sweeter taste and significantly influence overall flavour intensity, which aligns with consumer preferences in both commercial and domestic tomato cultivars [7,8,9]. The °Brix can be easily measured by refractometer, but estimating it solely based on maturity varies with the cultivar [10]. This measurement is particularly valuable in the food industry for quality control, ensuring consistency in products such as wines and sauces where sugar content is critical for taste and preservation [11].

Lycopene, a potent antioxidant and the primary carotenoid giving ripe tomatoes their red hue, has been extensively studied for its health benefits. A plethora of research [12,13,14,15] links lycopene intake to a reduced risk of chronic diseases like cardiovascular diseases and cancer. Beyond its health attributes, lycopene also influences consumer acceptance, with its content varying mainly due to factors like the tomato variety and environmental conditions including temperature, light, and water supply [16,17]. While the lycopene content can be roughly estimated based on fruit colour [18,19], precise measurements require more complex and costly laboratory analyses [20,21,22].

The colour and uniformity of tomato fruit are fundamental factors that consumers prioritize when assessing fruit quality. This visual attribute serves as the main determinant in tomato-purchasing decisions, as the ever-evolving shade of tomatoes (transitioning from green to deep red or even yellow based on the cultivar) act as vital visual cues. These cues subsequently influence consumer selection, quality evaluations, and market dynamics [23,24]. Consumers often associate specific colours with superior taste, higher nutritional value, and freshness. In general, fruit colour can be measured by visual analysis or different instrumental methods such as colorimetry, spectrophotometry, or a computer vision system. In this context, the chromaticity ratio (a/b ratio) derived from colorimetric data provides a quantifiable measure of the tomato’s colour balance, offering an objective method to assess the shift in hue as tomatoes ripen, which is crucial for quality control and breeding programs [25].

As the global population expands, the imperative to ensure consistent and high-quality tomato yields becomes even more paramount. This challenge is magnified by the uncertainties of climate change, which introduces threats such as droughts and rising temperatures, emphasizing the need for innovative agricultural approaches [26,27]. Concurrently, the technological advances of the digital age are furnishing the agricultural sector with expansive datasets derived from a myriad of sources. As farmers globally not only harvest crops but also glean invaluable data from their fields, there is a growing potential to harness this information to refine crop and management strategies [28]. A key objective in agriculture is to decrease production costs without compromising yield or quality [29]. Advancements in computer science have popularized machine learning (ML) techniques, which utilize features extracted from these datasets [30]. Such ML-driven insights can potentially revolutionize farming practices, making them more efficient and sustainable as highlighted in studies [31,32].

Two techniques have prominently emerged as viable contenders for agricultural data processing: extreme gradient boosting (XGBoost) and artificial neural networks (ANNs). XGBoost is a highly efficient gradient boosting framework, excelling in both classification and regression tasks [33,34]. It stands out as an advanced gradient boosting decision tree algorithm. Recognized for top performance, XGBoost is an open-source boosted tree toolkit, appreciated for its ability to combine multiple tree models into a powerful learning framework. Its proficiency in handling large-dimensional datasets, especially in gene expression research, highlights its significance [35,36,37,38]. Concurrently, ANNs have gained widespread recognition in the deep learning domain for their ability to process high-dimensional data and extract meaningful features [30,39]. These features offer transformative insights, potentially reshaping agricultural practices towards sustainability. Particularly in the field of remote sensing, ANNs are routinely employed to forecast vegetation parameters and crop yields, as demonstrated in studies [40,41,42]. However, the deployment of ANNs presents certain challenges, such as optimizing the number and size of hidden layers, determining the appropriate learning rate, the need for expansive training datasets, and confronting issues like overfitting.

Thus, in the backdrop of these advancements, this study embarks on a dual-pronged approach, harnessing the strengths of both XGBoost and ANNs to predict the quality metrics of tomatoes such as Brix, lycopene content, and fruit colour, the latter being quantified through the a/b ratio in the Hunter Lab colour space. By comparing their performances on a multi-year and multi-location dataset, this study aims to highlight the predictive capabilities of XGBoost and ANN. This will provide crucial insights for upcoming breeding initiatives and will make progress in ensuring tomato quality, especially in the face of growing challenges.

## 2. Materials and Methods

### 2.1. Dataset Description

In this study, a comprehensive dataset was utilized encompassing physicochemical characteristics and environmental factors across a diverse selection of tomato cultivars over five consecutive growing seasons from 2017 to 2021. The dataset included observations of 48 cultivars and 28 locations (Loc) within Hungary.

The selection and distribution of cultivars varied annually, with 25 cultivars at 7 locations in 2017, 22 cultivars at 18 locations in 2018, 27 cultivars at 19 locations in 2019, 27 cultivars at 18 locations in 2020, and 26 cultivars at 15 locations in 2021. This variability provided a rich dataset for analysing tomato quality traits under diverse environmental conditions. For each cultivar–location combination within a given year, multiple measurements were conducted on a random sample selection after harvesting on the same day to assess the quality traits of the tomatoes, ensuring the robustness of the dataset. A total of 28,747 individual measurements were recorded for each of the three main variables of interest which were the °Brix (denoting water-soluble solids content (Brix)), lycopene concentration, and fruit colour (quantified through the a/b ratio as a measure of colour balance in the Hunter Lab colour space).

To understand the impact of meteorological factors on tomato cultivation, meticulous records were analysed over growing seasons covering various climatic factors. These records were sourced from the Operational Drought and Water Scarcity Management System in Hungary (General Directorate of Water Management, Budapest, Hungary). This database provided a comprehensive overview of the conditions for each growing season, defined specifically as the period from 30 May to 30 August of each year, which is the favourable and usual growing period for tomatoes in Hungary, covering mostly the period from intensive vegetative growth to harvest. The number of days with temperatures between 21 °C and 27 °C (T21_27) was noted, as this range is optimal for tomato growth. Total precipitation (TotPrecip) during the growing season and the number of rainy days (RainDays) were recorded to understand moisture availability. Additionally, the average relative humidity (AvgRH) was monitored to assess the overall moisture content in the air. The number of days with relative humidity within the 40% to 70% range (RH40_70) was also tracked, being the ideal range for tomato cultivation. Furthermore, instances of high humidity were observed, specifically days when the average daily relative humidity exceeded 90% (RH_90+), as such conditions could adversely affect plant health. Alongside these climatic factors, the soil type (SoilTyp) at each location was classified according to the USDA soil classification system.

### 2.2. Measurement of Tomato Quality Traits

The physicochemical properties of tomatoes were assessed using state-of-the-art automated stations. Brix was measured by the SV01 from the Maselli Misure Quality Station (2020 Maselli Misure S.p.A, Parma, Italy), which first processed the tomatoes into juice followed by an automatic refractometric analysis to determine the water-soluble solids content, presented on a temperature-compensated scale with a range from 0 to 10 Brix and accuracy within ±0.15 Brix, adhering to the nD/Bx [43] standard. Lycopene content was quantified via an automated spectrophotometric analysis, reporting concentration levels in mg/100 g with measurement limits of 0 to 80 mg/100 g, an accuracy up to 0.5 mg/100 g, and a repeatability ±0.25 mg/100 g. Additionally, fruit colour was assessed through spectrophotometric analysis measuring the colorimetric coordinates L, a, b, from which the chromaticity ratio (a/b ratio) was derived to evaluate the balance between red and yellow hues, with a repeatability for X, Y, Z coordinates less than 0.07, ensuring consistency in the colour assessment of the tomatoes.

### 2.3. Data Preprocessing

The dataset underwent several preprocessing steps to ensure data quality and facilitate exploratory analysis. The initial preprocessing involved the transformation of categorical attributes such as ‘Loc’, ‘Cultivar’, and ‘SoilTyp’. Each of these attributes were transformed into one-hot encoded vectors to convert them into numeric representations suitable for ML algorithms [44,45]. Then, the dataset’s integrity was assessed by quantifying missing entries within each column. Missing values within numerical columns were imputed using the respective column’s mean, while those in categorical columns were replaced with the mode. This approach helped maintain the original distribution of the data and minimize the distortion introduced by imputation. After clean-up occurred, an in-depth exploration into the relationships between the different variables was conducted using a correlation matrix visualized on a heatmap, utilizing the seaborn library.

### 2.4. Machine Learning Models

#### 2.4.1. XGBoost Model

The XGBoost model is known for its efficiency in handling missing values and evaluating feature importance based on gradient-boosted decision trees. This model iteratively refines predictions by adding trees that minimize error [34]. To prepare the dataset for time series predicting, lag features for the ‘Predicted Variable’ (i.e., Brix, lycopene, a/b ratio) column were engineered, considering lag values from the previous one- to three-time steps. A rolling mean (moving average) feature was computed for the ‘Predicted Variable’ column, with a window of three time points to capture temporal patterns and to smoothen out short-term fluctuations [46]. The dataset was split into training and test subsets, employing a fivefold time series split method, partitioning the dataset into five sequential time-based segments. Each segment is utilized once as the test set, while all previous segments form the training set. This approach enables iterative training and validation of the model on distinct portions of the dataset, thereby maintaining the integrity of temporal sequences and avoiding leakages of future information during model training [47]. Each feature subset underwent standardization using the StandardScaler method, ensuring zero mean and unit variance. The XGBoost regression model was employed for the prediction task. The model’s hyperparameters were optimized through grid search coupled with threefold cross-validation. The hyperparameter grid encompassed various combinations of ‘n_estimators’, ‘max_depth’, ‘learning_rate’, ‘colsample_bytree’, and ‘gamma’ to minimize the squared error. Once the optimal hyperparameters were identified, the model was trained on the entirety of the training dataset and subsequently evaluated on the test set. The performance was assessed using the R-squared value, root mean squared error (RMSE) (1), and magnitude relative error (MRE) (2) where:

(1) $$\mathrm{RMSE}=\sqrt{\frac{1}{n}}\sum_{i=1}^{n}{\left(y_{i}-{\hat{y}}_{i}\right)}^{2}\mathrm{and}$$

(2) $$\mathrm{MRE}=\frac{\left|y_{i}-{\hat{y}}_{i}\right|}{\left|y_{i}\right|}$$

$n^{\;}$ is the number of observations in the dataset,
$y_{i}$ is the actual value for the *i*-th observation,
${\hat{y}}_{i}$ is the predicted value for the *i*-th observation.

#### 2.4.2. ANN Model

Artificial neural networks (ANNs), inspired by the human brain’s neural network, excel in modelling complex non-linear data relationships [45]. For the ANN model, data was sorted chronologically based on the ‘Year’ column. To capture potential temporal patterns, lag features were generated for the ‘Predicted Variable’ measurements spanning three previous time points. Additionally, a three-time point rolling average was computed to smoothen short-term fluctuations. The architecture of the model was determined through hyperparameter tuning, which included the number of neurons, dropout rates, and learning rates [48]. The network featured two hidden layers with a variable number of units, dropout layers for regularization, and an output layer for predictions. A random search, complemented by early stopping based on validation loss to prevent overfitting, facilitated systematic hyperparameter exploration. The data was split into training and test subsets using a fivefold time series split method, partitioning the dataset into five sequential time-based segments ensuring a chronological division and preventing future data leakage during the training process. Both training and test datasets were standardized to have zero mean and unit variance using StandardScaler. The trained ANN was then evaluated on the test set, with model performance evaluated using the R-squared value, RMSE, and MRE.

### 2.5. Feature Importance Analysis with SHAP

The SHAP (shapley additive explanation) value analysis, developed by Lundberg and Lee [49], was utilized to highlight the impact of individual features on the predictions of both XGBoost and ANN models. SHAP values measure each feature’s contribution to the prediction by assessing their marginal contribution across all possible feature combinations. For the XGBoost model, following optimization, SHAP analysis was conducted on features including ‘Loc’, ‘Cultivar’, ‘SoilTyp’, ‘AvgT’, ‘T21_27’, ‘TotPrecip’, ‘RainDays’, ‘AvgRH’, ‘RH40_70’, and ‘RH_90+’. The data was standardized using the StandardScaler method before computing the SHAP values for the training set, thus showcasing the average contribution of each feature [50]. In the case of the ANN model, the training data was adapted to be compatible with the SHAP library, employing the GradientExplainer method to compute SHAP values for the same features. For both models, categorical features such as ‘Loc’, ‘Cultivar’, and ‘SoilTyp’ required aggregation to assess their collective importance. SHAP summary plots were generated to visualize the relative importance and effect of each feature. These plots employed a dot plot format, where the x-axis represented the magnitude of SHAP values, and the y-axis represented the features. A dual-colour scheme was used, with red and blue indicating high and low feature values, respectively, illustrating the directional influence of each feature on the model predictions. The observed differences in the SHAP graphs between the XGBoost and ANN models can be primarily attributed to their intrinsic architectural differences and the specific methods used for SHAP value calculation. The XGBoost model operates within a gradient boosting framework and utilizes decision trees, facilitating a more straightforward computation of SHAP values by assessing the impact of each feature across an ensemble of trees. In contrast, the ANN model, comprising a complex network of neurons with non-linear activations, necessitates the use of approximation methods such as the SHAP. GradientExplainer makes the calculation of SHAP values more intricate. This complexity contributes to the variations observed in the visual representations of feature importances in the SHAP graphs for each model.

## 3. Results

### 3.1. Correlation Heatmap

**Figure 1:**
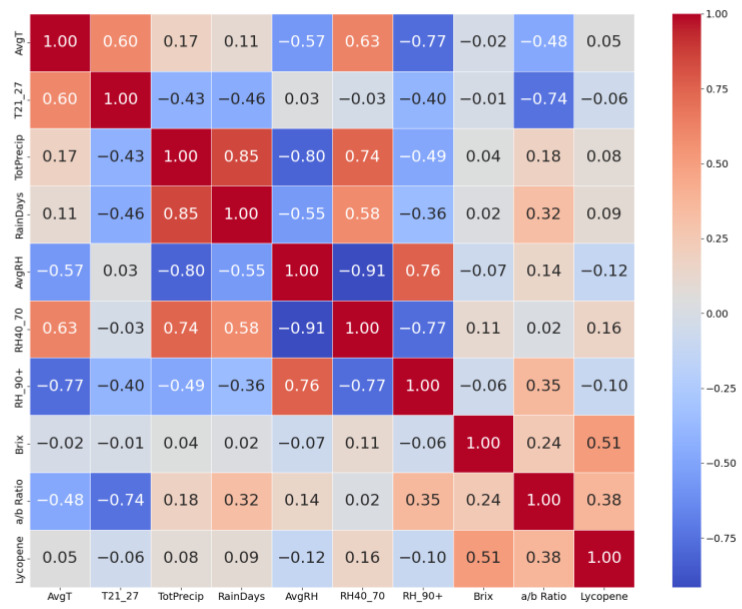
Correlation heatmap of Brix, a/b ratio, lycopene and key climatic variables. Hues of red and blue represent strength of correlation as it is visualized on the legend (n = 28,474).

The generated correlation heatmap offers a comprehensive insight into the linear relationships between the climatic variables, Brix, lycopene and a/b ratio (Figure 1). The intensity and direction of relationships are visually represented through a spectrum ranging from cool blue for negative correlations to warm red for positive ones, a method validated by Waskom [51]. Notably, the heatmap reveals a significant positive correlation between ‘AvgT’ (average temperature) and ‘T21_27’ (number of days with temperatures between 21 °C and 27 °C), suggesting that higher average temperatures during growing seasons often correlate with an increased number of days in the optimal temperature range for growth. Moreover, ‘TotPrecip’ (total precipitation) and ‘RainDays’ (number of rainy days) show a strong alignment, underscoring the intuitive link between increased rainy days and higher total precipitation, a key factor in agricultural water resource management and irrigation strategies. Conversely, an inverse relationship is observed between ‘AvgRH’ (average relative humidity) and ‘RH40_70’ (days with 40% to 70% humidity), indicating that seasons with higher overall humidity tend to have fewer days within the ideal humidity range for cultivation. The ‘a/b ratio’ also demonstrates notable correlations with several climatic parameters. All eight meteorological variables were incorporated as independent factors in our predictive models, aiming to provide comprehensive insights into the influences on fruit quality and yield.

### 3.2. Model Performance

#### 3.2.1. Brix

**Figure 2:**
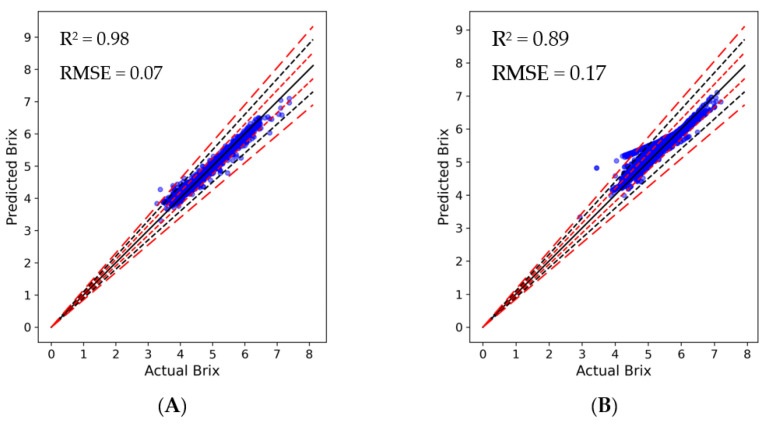
Actual vs. predicted Brix utilizing the (**A**) XGBoost and (**B**) ANN models. Black solid line indicates perfect prediction, meaning that y = x. Red short-dashed lines, black dashed lines, and red long-dashed lines indicate ± 5, 10, and 15% deviation from the y = x line, respectively (n = 28,474).

The developed algorithms exhibited a high degree of accuracy when estimating the Brix values (Figure 2). The XGBoost model yields an impressively robust R^2^ value of 0.98 and low RMSE of 0.07. Such results not only vouch for the XGBoost algorithm’s capability but also highlight the significance of the chosen features in predicting Brix values from other climatic and quality variables. On the other hand, the ANN model resulted in an R^2^ of 0.89 and RMSE of 0.17, marking its good performance in intricate predictive modelling scenarios. The presented scatter plots from the two distinct models provide insights into their performance efficacy in predicting Brix values. Both plots display a significant concentration of data points around the black line representing x = y, highlighting the commendable accuracy of both models. For the XGBoost model and the ANN model, the percentage of predictions deviating less than 5% are 97% and 89%. Those deviating between 5% and 10% were 2.6% and 8.4%, respectively, and those deviating between 10% and 15% were 0.4% and 1.4%. Those deviating more than 15% are 0.06% and 1.12%. It is noteworthy that a predominant cluster of data points for both models lie within the 5% error margin, signifying that the model predictions are not only accurate but also consistent. These statistics underscore the models’ competence in closely estimating the actual water-soluble solid content, despite some error margins which can be expected in predictive modelling.

**Figure 3:**
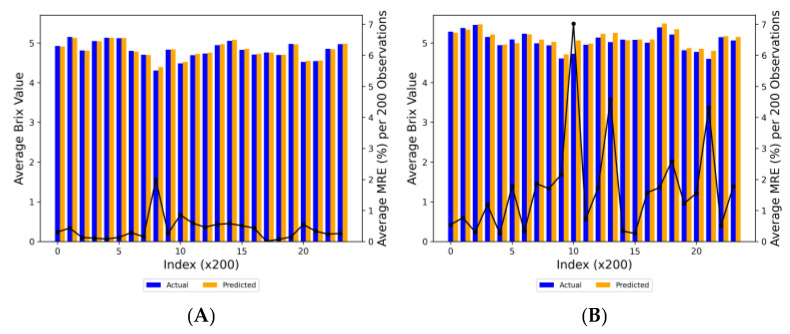
Comparison of MRE (black line) of actual (blue bars) vs. predicted (orange bars) Brix values per 200 observations (Index × 200) for (**A**) XGBoost and (**B**) ANN models.

The MRE graph in Figure 3 provided a visual assessment of the prediction errors made by the XGBoost and ANN models in estimating Brix values. According to Figure 3A, the MRE for the XGBoost model was as low as approximately 0.25% in some intervals, indicating high predictive accuracy. However, it reached upwards of 2% in others, suggesting a reasonable predictive performance overall. On the other hand, the second graph demonstrated the MRE for the ANN model, which ranged significantly from approximately 0.5% to nearly 7%. While both models showed areas of agreement between actual and predicted Brix values, the ANN model exhibited higher variability in prediction accuracy. This variability suggested that, in this specific application, the XGBoost model might have offered more consistent predictions compared to the ANN model.

#### 3.2.2. Lycopene

**Figure 4:**
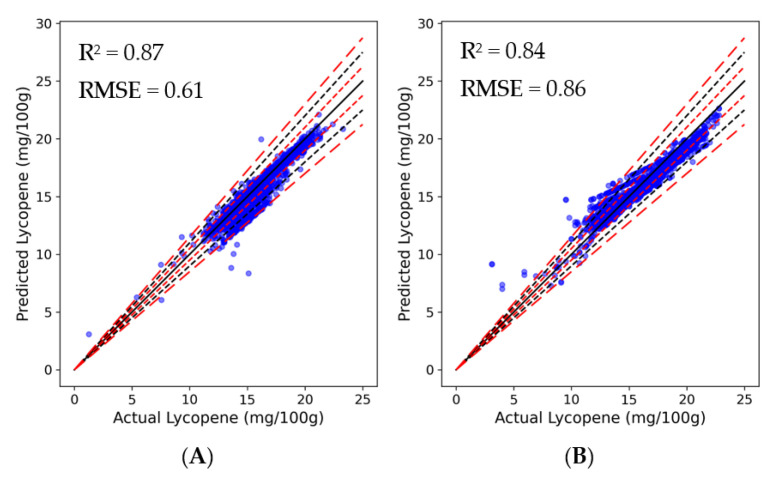
Actual vs. predicted lycopene utilizing the (**A**) XGBoost and (**B**) ANN models. Black solid line indicates perfect prediction, meaning that y = x. Red short-dashed lines, black dashed lines, and red long-dashed lines indicate ± 5, 10, and 15% deviation from the y = x line, respectively (n = 28,474).

It is represented in the graphs that a high degree of correlation was exhibited with predicted and actual lycopene contents for both algorithms (Figure 4). The XGBoost model yielded an R^2^ value of 0.87 and a RMSE value of 0.61, accounting for 87% of the variance in observed lycopene content. In contrast, the ANN model had an R^2^ of 0.84 and a RMSE of 0.86, attesting to its substantial explanatory capability. While both models exhibited commendable accuracy in predicting the lycopene content, minor inconsistencies were observed. The line representing ideal prediction, where predicted values coincide with actual measurements, serves as a benchmark for accuracy. It was revealed that a significant proportion of predictions from both models lie within the 10% deviation margin, underscoring their precision. More specifically, for the XGBoost model and the ANN model, the percentage of predictions deviating less than 5% were 84.55% and 86.45%, respectively, and predictions that deviated between 5% and 10% were observed to be 10.31% and 10.28%, respectively. Those that fell between 10% and 15% deviation were 4.81% and 1.96%, and finally, predictions that deviated more than 15% were at 0.34% and 1.31%.

**Figure 5:**
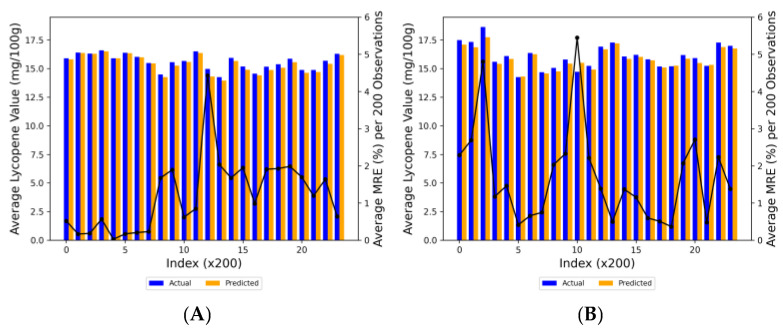
Comparison of MRE (black line) of actual (blue bars) vs. predicted (orange bars) lycopene values per 200 observations (Index × 200) for (**A**) XGBoost and (**B**) ANN models.

The MRE graph in Figure 5 revealed fluctuations in prediction accuracy across the dataset. Comparatively, the XGBoost model demonstrated a more stable performance, with most data groups maintaining an MRE below 4%, suggesting generally robust predictive accuracy. On the other hand, the ANN model, as depicted in Figure 5B, exhibited higher variability in its MRE, oscillating across different values and suggesting varying degrees of predictive accuracy. Notably, some segments exhibited a relatively high MRE, peaking just below 6%. The bar representations of actual versus predicted lycopene values in both graphs were closely aligned, indicating reasonable predictive capabilities. The XGBoost model presented slightly superior performance in terms of consistency and reduced error.

#### 3.2.3. a/b ratio

**Figure 6:**
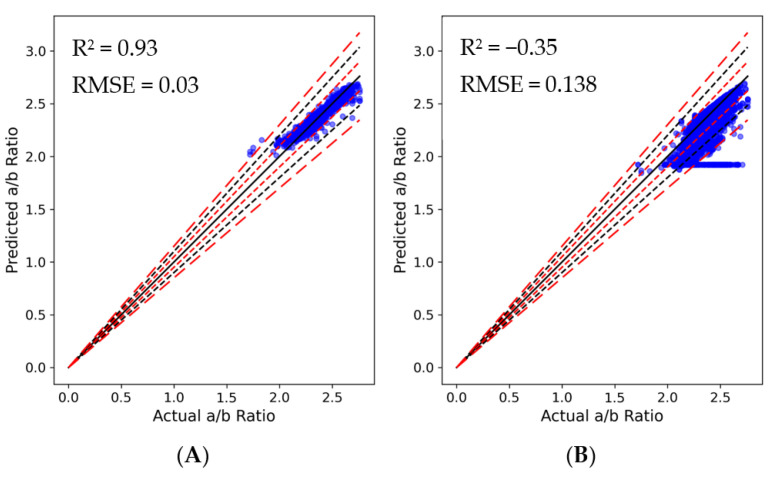
Actual vs. predicted a/b ratio utilizing (**A**) XGBoost and (**B**) ANN models. Black solid line indicates perfect prediction, meaning that y = x. Red short-dashed lines, black dashed lines, and red long-dashed lines indicate ± 5, 10, and 15% deviation from the y = x line, respectively (n = 28,474).

The XGBoost model demonstrated a high degree of accuracy, achieving a R² value of 0.93 and a RMSE of 0.03, indicating a strong fit to the data (Figure 6A). In contrast, the ANN model yielded a higher RMSE of 0.138. While this suggested a reasonable proximity of predictions to actual observations, the model’s negative R² value of −0.35 indicated a poor fit to the dataset. This finding suggested that either the current ANN model was not optimal for this dataset, or there were underlying issues with either the dataset or its processing. In terms of prediction deviation, 99.45% of predictions had been within 5% of the actual values for the XGBoost model, while only 0.42%, 0.13%, and 0.00% had deviated by 5–10%, 10–15%, and over 15%, respectively. This indicated a high level of accuracy for most predictions. On the other hand, the ANN model had shown larger deviations: 81.29% of predictions had been within 5%, and 13.32%, 2.93%, and 2.47% had deviated by 5–10%, 10–15%, and over 15%, respectively. Notably, the ANN model had displayed significant deviations beyond the ±5% and ±10% margins (Figure 6B), suggesting areas of unreliability. It is worth noting that despite the moderate correlation observed in the ANN model indicating a positive linear relationship between observed and predicted values, the negative R² value pointed to its failure in adequately fitting the variance in the data. This discrepancy underscored the importance of comprehensive evaluation metrics in model assessment. The RMSE of 0.138, while seemingly small, was significant if the dependent variable in the dataset exhibited low variability. This magnitude of RMSE reflected the fact that ANN model’s predictions were, on average, 0.138 units away from the actual values, leading to consistent and notable inaccuracies. Thus, the practical utility of the ANN model in this context was limited, as evidenced by its negative R² value, despite a moderate correlation.

**Figure 7:**
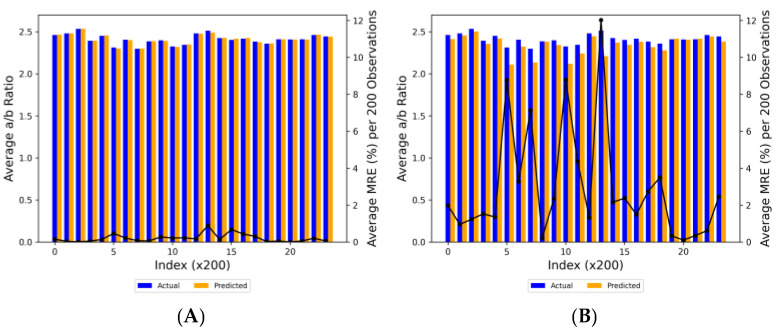
Comparison of MRE (black line) of actual (blue bars) vs. predicted (orange bars) a/b ratio values per 200 observations (Index × 200) for (**A**) XGBoost and (**B**) ANN models.

In our analysis, the XGBoost model demonstrated satisfactory predictive performance. Its MRE fluctuated but remained relatively low, peaking slightly above 0.8% (Figure 7A). In contrast, the ANN model exhibited significantly greater variability in its predictions. The MRE of the ANN model reached as high as approximately 12%, indicating that, on average, its predictions deviated by a maximum of 12% from the actual values. Although the bar representations of both actual and predicted a/b ratio values in the two graphs suggested a decent level of predictive accuracy, the XGBoost model markedly outperformed the ANN model in terms of prediction fidelity and consistency.

### 3.3. SHAP

#### 3.3.1. Brix

**Figure 8:**
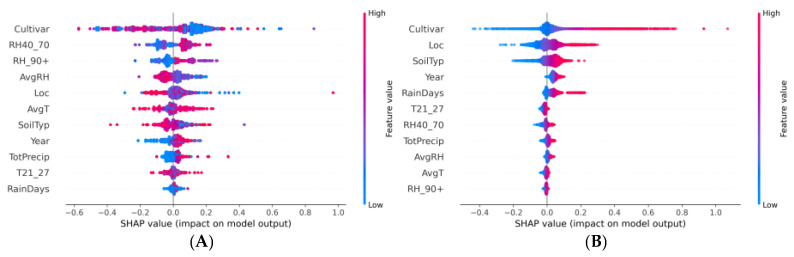
SHAP summary plot of Brix prediction for (**A**) XGBoost and (**B**) ANN models.

Noticeable differences were observed in the importance of features and their effects on the models’ predictions as a result of the conducted comparative analysis of the SHAP summary plots for the XGBoost and ANN models (Figure 8). The most important difference between the SHAP plots of the two ML model was that positive feature values contributed to mainly positive SHAP values in the ANN model, but were sorted differently for the XGBoost. The ‘Cultivar’ feature was paramount in the XGBoost model, displaying a broad range of SHAP values that are both positive and negative, indicating a robust association between certain cultivars and elevated Brix levels. This suggested the significance of genetic attributes in enhancing water soluble solids content. The features related to humidity such as ‘RH40_70’ and ‘AvgRH’ showed a substantial spread of SHAP values across the x-axis, suggesting variable effects on Brix prediction, where both low and high relative humidity levels could either positively or negatively impact the accumulation of water-soluble solids in fruits, contingent upon other interacting variables. In contrast, in the ANN model the plot revealed a consistent pattern: higher feature values are invariably associated with positive SHAP values, while lower feature values correspond to negative SHAP values. This suggests a monotonic behaviour where the magnitude of a feature’s value is directly proportional to its impact on the output of the model. The ‘Cultivar’ feature demonstrated a more uniform effect across the entire dataset, with a tendency toward positive contributions, reflecting its significant and consistent influence on the model’s prediction of the Brix. Similarly, the SHAP values for ‘Loc’ and ‘SoilTyp’ indicate that geographical location and soil type are influential factors in predicting Brix levels, with higher and lower values of these features consistently impacting the model’s output. The variable ‘Year’ also emerged as a significant temporal factor in the ANN model, potentially capturing the effects of varying climatic conditions across years, indicative of the model’s capability to assimilate temporal dynamics into its predictive mechanism. The SHAP analysis showed that the XGBoost model attributed more importance to ‘AvgT’ than to ‘TotPrecip’. Contrastingly, the effect of ‘TotPrecip’ on the prediction of Brix was important in the ANN model. However, the ways in which these factors influenced Brix predictions in each model differed, possibly reflecting inherent differences in data assumptions and the models’ strategies for integrating features.

#### 3.3.2. Lycopene

**Figure 9:**
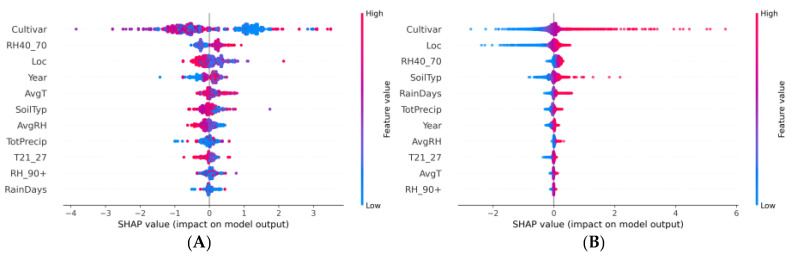
SHAP summary plot for lycopene prediction for (**A**) XGBoost and (**B**) ANN models.

The SHAP summary plots for the XGBoost and ANN models provided valuable insights into the determinants of lycopene content in tomato fruits (Figure 9). The analysis of the XGBoost model revealed that the ‘Cultivar’ and ‘RH40_70’ features had a significant impact on the model’s predictions of lycopene content. The ‘Cultivar’ feature showed a wide spread of SHAP values, indicating that different cultivars had varying levels of influence on the lycopene prediction. This suggested a complex, potentially non-linear relationship with the target variable. Variable ‘RH40_70’ showed a more concentrated range of SHAP values, suggesting a consistent but less influential effect on the model’s predictions. Other features were represented with SHAP values clustered closer to the centre, implying a more moderate impact on the lycopene content prediction. For the ANN, the ‘Cultivar’ feature exhibited the most substantial influence on the model’s output with a broad spread of dots, indicating that the influence was more positive than negative. This implied a complex interplay where certain cultivars could have had a substantial impact, either augmenting or diminishing the potential lycopene content determined by genetic background. Although the general directionality of feature values and their impact on the model’s predictions might have suggested a monotonic pattern, the spread and distribution of the SHAP values did not necessarily imply a linear relationship but rather a consistent pattern recognized by the neural network where certain features were favourable for lycopene production. The colour gradient added another layer of interpretability. For instance, the XGBoost plot showed that both high and low values of ‘AvgT’ did not exhibit simple linear relationships with lycopene. Instead, its impact was nuanced, with both high and low values influencing predictions in both positive and negative directions. This complexity may have mirrored how biological processes formed agricultural crops in response to environmental factors. Additionally, temporal trends reflected in the ‘Year’ feature’s SHAP values could have pointed to evolving agricultural practices or climatic shifts over time, further highlighting the multifaceted nature of lycopene biosynthesis.

#### 3.3.3. a/b ratio

**Figure 10:**
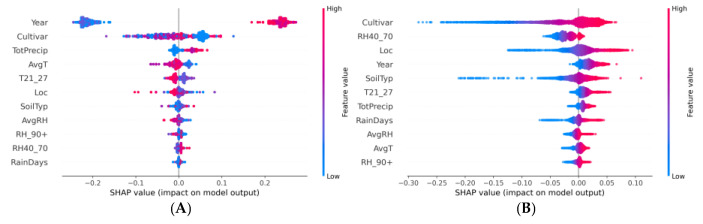
SHAP summary plot for a/b ratio prediction for (**A**) XGBoost and (**B**) ANN models.

Examining the SHAP summary plots of the two ML models that had been designed to predict tomato fruit colour values (particularly the a/b ratio), distinct patterns of feature influence had emerged (Figure 10). The ‘Year’ feature in the XGBoost model had exhibited a high distancing of SHAP values, with clusters on both the positive and negative sides of the zero line, indicating a variable influence on the model’s prediction with some years contributing to an increase and others to a decrease in the predicted a/b ratio. The ‘Cultivar’ feature exhibited a unidirectional effect, with a pronounced aggregation of its SHAP values on the positive side, indicating a uniform contribution to the increase in the model’s predicted a/b ratio. Notably, this increase is predominantly associated with the lower encoded values of ‘Cultivar’, as indicated by the abundance of blue points. Conversely, ‘TotPrecip’ was predominantly associated with decreases in the a/b ratio, suggesting a positive relationship. For the ANN model, interpreting the SHAP values became more challenging due to the negative R^2^ score. The model had predominantly exhibited negative SHAP values for features such as ‘Cultivar’, ‘SoilTyp’, and ‘RH40_70’. These consistently downward predictions indicated that these features often reduced the predicted value compared to the model’s baseline. The dominance of negative SHAP values and the lack of variation in SHAP value direction, unlike the variability observed in the XGBoost model, raised concerns about potential overfitting, insufficient feature representation, or inadequate network architecture to capture the complexities of the dataset. Furthermore, the ANN’s poor performance metric, as highlighted by the negative R² score, implied that the model was less informative than a simple average of the target variable, suggesting that the model’s internal representations and learned weights did not generalize well to the data’s underlying structure.

## 4. Discussion

### 4.1. Correlation Heatmap

The correlation heatmap provided an invaluable visual summary of the intricate interrelationships among climatic variables, Brix, lycopene, and a/b ratio in tomato fruits. The strong positive association between ‘AvgT’ and ‘T21_27’ underscored the synchronicity of average seasonal temperatures with the frequency of days experiencing temperatures between 21 and 27 °C. This relationship is pivotal, as temperatures within this range were known to be conducive for the optimal growth of tomato plants and could influence various biochemical processes, including the synthesis of sugars and pigments [52]. Close alignment was found between ‘TotPrecip’ and ‘RainDays’, affirming the notion that seasons with more accumulated rainfall were characterized by a higher number of rainy days. Excessive rainfall, especially during the fruit development stage, could influence fruit texture and water content, and could even lead to conditions such as fruit cracking [53]. As was expected, an inverse correlation was observed between ‘AvgRH’ and ‘RH40_70’ and could be indicative of specific climatic patterns affecting the impact of certain stresses. A season with consistently high humidity might have had fewer fluctuations, resulting in fewer days with humidity levels within the 40% to 70% range. Such patterns could influence plant transpiration rates, nutrient uptake, and susceptibility to certain diseases [54]. High humidity levels might reduce transpiration rates, leading to an accumulation of sugars in the fruit, thereby elevating the Brix values [55], however, no correlation was found between Brix and RH_90+. It is well-established that external factors can modulate the synthesis of pigments and antioxidants in tomatoes [56,57]. By contrast, there was no significant correlation revealed between climatic factors and Brix or lycopene content. Instead, the a/b ratio correlated significantly with T21_27, and moderate relationships were indicated with AvgT, RainDays, and RH_90+.

### 4.2. Model Performance

#### 4.2.1. Brix

The prediction of water-soluble solids content, which is an important quality trait for the food and beverage sector, was effectively handled by our ML models [11]. The XGBoost model demonstrated slightly superior performance, attributed to its gradient boosting mechanism which effectively handle linear and non-linear relationships, missing values, outliers, and diverse data types. Conversely, the ANN showcased robustness in capturing intricate patterns in multi-dimensional data. Its performance in predicting Brix values, though substantial, suggested limitations in capturing certain complexities, unlike XGBoost. This research built upon previous findings such as Silva et al. [58], who used a global climate model and highlighted the significant impact of extreme climatic conditions (like increased heat and dry stress) on tomato quality. These conditions were crucial factors that could potentially enhance the accuracy of ML predictions. Complementing this, Zuo [59] demonstrated the use of visual datasets in tomato quality grading using ML and image processing, and Égei et al. [60] revealed the efficacy of VIS-NIR spectroscopy in determining soluble solids content applying partial least square regression (PLSR) model obtaining R^2^ of 0.72 and 0.88 for calibration and validation, respectively. Notably, our models, derived from climatic and environmental data using more cost-effective methods, amplified their potential for broader, non-destructive applications. The significance of this approach was highlighted by comparing it with earlier works. For instance, Ecarnot et al. [61] reported a R² of 0.86 using a portable VIS-NIR spectrometer for the rapid assessment of tomato Brix, whereas our refined ML approaches demonstrated greater precision. Additionally, the non-destructive Brix prediction model by Gomes et al. [62,63] showed a R² of 0.95 and RMSE of 1.34 using PLSR, and a R² of 0.91 and RMSE of 1.36 using principal component analysis (PCA), underscoring the enhanced efficacy of our ML methods (especially regarding RMSE). Ultimately, the significant aggregation of predictions within the 5% error margin for both models highlighted their practical value for predicting tomato quality in relation to climatic conditions, demonstrating their potential for aiding in long-term agricultural planning and ensuring consistent product quality over time. The minimal inaccuracies observed, particularly for values scattered in brackets with higher error, further attested to the robustness and reliability of these models.

#### 4.2.2. Lycopene

Lycopene content is a pivotal component in determining the nutritive and organoleptic qualities of tomatoes. In our analysis, both the XGBoost and ANN models demonstrated a significant positive correlation between the predicted and actual values of lycopene content, with R² values of 0.87 for XGBoost and 0.84 for ANN. These figures indicated a strong correlation, aligning with previous studies that highlighted the effectiveness of ML in agricultural data analysis [64,65,66]. The XGBoost model, traditionally renowned for handling structured/tabular data [34], showed a slightly better performance with a RMSE of 0.61, compared to the ANN model’s RMSE of 0.86. This can be attributed to its scalability and capability of handling various types of prediction problems, including its resilience against overfitting and ability to implicitly handle missing values. Conversely, ANNs are known for their versatility in handling complex, non-linear data patterns [67,68]. Although the ANN model here showed a marginally lower precision than XGBoost, it is important to consider that its performance can be influenced by factors such as architecture design and the number of layers. The high percentage of predictions within 10% deviation from actual values (84.55% for XGBoost and 86.45% for ANN) underscored the practical applicability of these models in precision agriculture, particularly for quality control and breeding programs [69]. Liu et al. [70] utilized methods including partial least squares (PLS), least squares-support vector machines (LS-SVM), and back propagation neural network (BPNN) to predict lycopene content from spectral data, reporting R² values of 0.50, 0.91, and 0.93, respectively. Similarly, Sharma et al. [71] used linear multivariate regression (LMVR) to predict lycopene content in tomatoes using physicochemical attributes, achieving a R² of 0.7. These findings highlighted the enhanced capabilities of modern XGBoost and ANN models in accurately predicting lycopene content. Despite the impressive performance of our models, it is crucial to acknowledge that all predictive tools are subject to inherent limitations. Factors such as sample diversity, experimental conditions, and algorithmic assumptions can affect their precision. Ultimately, both the XGBoost and ANN models demonstrated significant potential for predicting lycopene content. However, due to its simplicity and proven track record, the XGBoost model emerges as the more favourable choice in our study.

#### 4.2.3. a/b ratio

In our study, the comparison between XGBoost and ANN models in predicting the a/b ratio in tomato cultivars offers significant insights. The XGBoost model, known for its gradient boosting framework and ability to manage varied datasets [34], demonstrated a substantial advantage. It not only showed higher accuracy, as evidenced by an impressive R² value of 0.93 and a minimal RMSE of 0.03, but also greater consistency in predictions. This indicates the model’s robustness in capturing the complex interplay of climatic and soil parameters, benefiting from its adaptability and regularized boosting technique. Conversely, the ANN model’s performance was not satisfactory. It exhibited a negative R² value of −0.35 and a higher RMSE of 0.138, suggesting significant issues in its fit to the dataset and potential problems such as overfitting, inadequate training, or a mismatch in model complexity [45,72]. The negative R² value suggested that the model’s predictions were worse than a simple mean of the observed data, raising questions about its suitability for this application. Additionally, the discrepancy between RMSE and R² could be due to RMSE’s sensitivity to outliers, while R² reflects the overall variance explained [73]. Furthermore, the prediction deviation analysis underscored the XGBoost model’s reliability, with 99.45% of its predictions within a 5% margin of the actual values, demonstrating its utility for precision-dependent applications [34]. In contrast, the ANN model showed larger prediction deviations, with only 81.29% of predictions within the same margin, highlighting its limitations in high-precision applications [45]. The significance of RMSE in datasets with low variability becomes particularly noteworthy—even a seemingly small RMSE in the ANN model indicates consistent and notable inaccuracies [74]. Moreover, the ANN model’s negative R² value underlines a fundamental inadequacy, suggesting its inefficiency compared to even basic mean-based prediction models [73].

### 4.3. SHAP

Recognizing the critical role of interpretability in agricultural applications, our analysis was extended to SHAP value computations. The SHAP summary plots of the two ML models revealed the influence of different features on the prediction values. These plots served as interpretable visual aids that can elucidate the complex inner workings of these models, especially in a domain that requires a nuanced understanding of the interplay between multiple factors [75].

#### 4.3.1. Brix

Our analysis revealed distinguishing features between the XGBoost and ANN models in predicting Brix values in tomato fruits, aligning with previous research that highlights the sensitivity of ML models to feature selection and interaction [76]. The prominence of ‘Cultivar’ in the XGBoost model echoed the findings in [77,78] where it was reported that the genetic makeup of a cultivar as a decisive factor in fruit soluble solids content. The positive SHAP values associated with ‘Cultivar’ suggest that certain genetic characteristics may be key drivers of Brix levels, potentially offering a pathway for targeted breeding programs [79,80,81]. The variable impacts of relative humidity observed in our study are consistent with the results published by Shin et al. [82], which demonstrated the complex roles of relative humidity in tomato fruit development and ripening. Our findings suggest that not only the range but also the duration of specific humidity levels could be critical, warranting further investigation into their interactions with other environmental factors. In contrast, while ANNs are inherently equipped to model complex, non-linear interactions [83,84], the monotonic behaviour observed in the SHAP plot suggests that the model may be capturing more direct and additive relationships between features and the Brix for the given dataset. Such an observation suggests that the neural network has adapted to the dataset’s structure by identifying and leveraging what appears to be a straightforward linear association of features with the target variable. The distributions of SHAP values for ‘Loc’ and ‘SoilTyp’ underscore the potential for ANN models to discern subtle influences of edaphic and geographical factors, aligning with [85,86], which posit that soil characteristics could profoundly affect fruit quality. For instance, certain soil types may be consistently beneficial or detrimental to the dissolved sugar content, depending on their nutrient profiles or water retention capacities. The role of the ‘Year’ variable in capturing annual climatic variations provided an intriguing insight into the temporal dynamics affecting Brix levels. As suggested in [87] and [88], shifts in agricultural practices, adoption of new technologies, or even changing climate patterns can manifest in fluctuations in the quality and nutritional content of crops. The distinct influences of meteorological factors observed in our study add to a growing body of evidence that suggest weather conditions play a pivotal role in Brix levels, which is also supported by the comprehensive analysis of climate impacts on fruit nutrition value by Stewart and Ahmed [89]. While our results provided valuable contributions to predictive modelling in agriculture, they also emphasized the importance of considering the specific model’s interpretive framework. The differences in feature importance between the XGBoost and ANN models could reflect the indicate of fundamental differences in their data processing methodologies [90]. This underlines the importance of interpretability and reliability in ML models, especially in domains where decision making is closely tied to model outputs [91].

#### 4.3.2. Lycopene

The XGBoost model, except for the ‘Cultivar’ and ‘RH40_70’ features, demonstrated a balanced feature influence with tight SHAP value clustering, suggesting a nuanced consideration of feature contributions, akin to findings by Lundberg and Lee [49] on interpretable ML models. Notably, the ‘Cultivar’ variable had stood out as a significant determinant with a complex and non-linear influence on lycopene content, in line with the research of Lundberg and Lee [92], which reported the subtleties of genetic factors in crop quality predictions. Furthermore, the finding was in agreement with the work of Bineau et al. [93], documenting the genetic diversity among tomato cultivars and its impact on the accumulation of secondary metabolites. However, the broad distribution of SHAP values for the ‘Cultivar’ feature within the ANN model likely signifies the model’s ability to capture complex, non-linear interactions between this feature and the lycopene, an aspect that mirrors the observations made by Wang et al. [94] regarding the capabilities of deep learning in capturing intricate biological phenomena. The spread of SHAP values for environmental features like ‘RH40_70’ and ‘Loc’ underscored the multifactorial nature of lycopene synthesis, as suggested in [95], emphasizing the critical roles of both genetic and environmental factors. This is further supported by [96,97,98], in which the influence of specific environmental conditions on lycopene synthesis and preservation was noted. The influence of the ‘AvgT’ on the lycopene content prediction potentially indicates adaptive physiological responses to environmental stresses, aligning with [99] on plant stress biology, where extreme temperature could be associated with either higher or lower lycopene content. Temporal variability in lycopene content, signified by the ‘Year’ SHAP values, could be indicative of the dynamic interplay between cultivation methods, environmental shifts, and plant genetics over time. This observation aligns with the longitudinal studies by Arah et al. [100], highlighting the evolutionary trajectories in agricultural practices and post-harvest handling techniques. While these insights were compelling, a potential risk of overfitting with the ANN model, as indicated by the extensive spread of SHAP values, must be acknowledged. Further validation with independent datasets, as recommended in [101], would be necessary to confirm the robustness of the findings. Additionally, integrating multi-omics data, as discussed by Kang et al. [102], could enhance the interpretability of the predictive models, offering a more holistic view of the factors influencing the lycopene content.

#### 4.3.3. a/b ratio

The observed variability in the SHAP values for the ‘Year’ feature within the XGBoost model aligns with previous research that indicates temporal dynamics can significantly affect agricultural outcomes [103]. The dispersion suggested that the impact of ‘Year’ on the a/b chromaticity ratio was not linear and may be influenced by other interacting factors, such as changing climate conditions or agricultural practices over time [104,105,106]. The ‘Cultivar’ feature’s consistent influence on increasing the a/b ratio, particularly at lower feature values, confirmed the importance of genetic factors in determining tomato fruit colour [107]. The positive pronounced aggregation of the SHAP values reflected a potentially strong genotype–phenotype relationship, which has been widely documented in crop quality traits [108]. In contrast, the ‘TotPrecip’ feature’s association with the a/b ratio may be indicative of the dilution effect of precipitation on fruit colour concentration, a finding that was supported by the work of Oh et al. [109], who noted that water availability could lead to the dilution of phytochemicals in fruits. Additionally, precipitation can modulate physiological processes in plants, impacting the synthesis and accumulation of pigments responsible for colour, which in turn affected the a/b ratio as was supported in [110]. The ANN model’s predominantly negative SHAP values and the accompanying negative R² score present a stark contrast to the XGBoost model and raise questions about ANN’s suitability for this task. This finding is particularly surprising regarding the increasing reliance on ANN models in precision agriculture [111]. The consistent underperformance, as indicated by the negative R² score, may be due to overfitting, which is a common challenge with ANN models [112]. It is suggested that the network architecture may have not been adequately optimized for the dataset. The lack of variation in the direction of SHAP values for the ANN model contrasted sharply with the XGBoost model and suggested that the former may not be capturing the true underlying data patterns. This discrepancy emphasizes the need for a thorough cross-validation and hyperparameter tuning process, which has been identified as a crucial step in model development [113,114]. Furthermore, the negative R² score suggests that the ANN model’s predictive power was worse than a naïve model, which would simply predict the average a/b ratio for all observations [115].

## 5. Future Work and Recommendations

To address limitations in our current models, a more comprehensive approach to data collection and diversity could be implanted. By incorporating a broader spectrum of climatic variables such as light intensity and quality and wind speed, we can provide a deeper understanding of environmental impacts on tomato quality [116,117]. Furthermore, by expanding the dataset to include a wider variety of tomato cultivars (like heirloom or more hybrid varieties), we could allow for a more robust analysis of genetic factors influencing Brix, lycopene, and a/b ratio [118,119].

Using advanced data preprocessing methods such as feature scaling normalization [101] and non-linear transformations [120] could significantly improve our model’s accuracy. Additionally, the incorporation of anomaly detection methods [121] could help in identifying and handling outliers, ensuring the reliability of the models.

For the ANN model, especially in predicting the a/b ratio, recalibration is needed. Investigating various neural network architectures like deeper networks or recurrent neural networks might help us capture temporal and complex interactions more efficiently [45]. Additionally, experimenting with different activation functions such as leaky rectified linear function (LReL) [122] or optimization algorithms [123] may also enhance the model’s performance. In the same way, for the XGBoost model, optimizing hyperparameters like the learning rate, tree depth, and the number of trees can improve its performance [34]. Exploring feature interaction constraints [124] could be useful for understanding complex data relationships better and improving the model’s performance.

In agricultural research (particularly predictive modelling) the exploration and implementation of diverse algorithms and methodologies hold significant potential. The idea of hybrid models, notably the combination of XGBoost and ANNs, could offer a promising research path. Such models could effectively integrate the feature interactions captured by tree-based algorithms with the complex pattern recognition abilities of neural networks. This approach aligns with ensemble techniques, as suggested by Shahhosseini et al. [125], where combining multiple model predictions such as a weighted ensemble of XGBoost and ANN enhances both stability and accuracy. Moreover, the concept of model stacking, introduced by Wolpert [126], involves using the outputs of XGBoost and ANN as inputs for a secondary model, possibly a simpler regression model, to enhance the accuracy of predictions further.

Deep learning is known for its capability to handle large and complex datasets, and stands as an effective strategy for capturing nonlinear interactions between environmental, genetic, and temporal factors. Convolutional neural networks (CNNs) for example, could be used to analyse satellite or field imagery in order to assess crop health and predict quality traits [127]. Additionally, recurrent neural networks (RNNs), especially long short-term memory (LSTM) networks, could be effective in modelling sequential data such as time-series climatic data to predict crop quality attributes [128].

Unsupervised learning algorithms are not only capable of analysis but also of evaluation. Clustering techniques like K-means or hierarchical clustering could give insights into sub-populations or environmental conditions within agricultural data, as indicated in [129]. PCA can help reducing dataset complexity, highlighting key features, and boosts model efficiency and interpretability as described in [130].

ML in agriculture plays a key role as decision-supporting tool, helping farmers in selecting appropriate cultivars and optimizing planting schedules by predicting important factors such as Brix and lycopene content. This aligns with the findings of Lobell and Gourdji [131], who highlighted the importance of predictive models in crop selection and agricultural productivity. Moreover, considering the influence of climatic variables, these models can assist in adapting farming practices to changing weather patterns. Tools developed from these models can predict the impact of anticipated climatic changes on crop quality, thereby aiding in the development of proactive strategies, a concept reinforced by Ray et al. [132] who emphasized the importance of climate-adaptive agricultural practices. Additionally, the substantial impact of climatic factors on tomato quality highlights these models’ potential in studying the broader effects of climate change on agriculture. Researchers and policymakers can use these models to project future trends in crop quality under various climate scenarios, aiding in forming mitigation strategies. This aspect is supported by Challinor et al. [133], who emphasized the importance of modelling in understanding climate change impacts on agriculture.

These findings highly valuable in the food industry as they can serve the development of non-destructive quality assessment tools, especially for assessing Brix content, which is essential for ensuring taste and quality [134]. Additionally, predictive models also play a crucial role in maintaining product consistency, a key factor for consumer satisfaction and brand reputation, by adjusting processing parameters, a point highlighted in [135]. Furthermore, the models from this study hold a promise in the potential application beyond tomatoes. They could advantage a deeper understanding and optimization of quality parameters across various agricultural products of other crops, as supported by Liakos et al. [64], in showcasing the diverse applications of ML in agriculture.

## 6. Conclusions

These findings underscore the superior predictive capabilities of the XGBoost model in the aforementioned scenarios and reveal limitations of the ANN model, especially in predicting a/b ratio. The SHAP summary plot analysis shows that both models effectively predict Brix values and lycopene content in tomatoes, but with different focal points. XGBoost emphasized the genetic makeup of cultivars and their interaction with environmental factors, whereas the ANN model captures complex genetic interactions and direct feature relationships. Additionally, our results highlighted the significant influence of temporal factors, particularly ‘Year’, on the a/b chromaticity ratio, suggesting a complex interplay with climatic conditions and agricultural practices. The limitations of the ANN model in this aspect, as evidenced by its negative SHAP values and R² score, underline the necessity of meticulous model selection, optimization, and validation in precision agriculture.

## Data Availability

Data available upon reasonable request and under certain conditions.
